# Concepts of Nanoparticle Dose Metric and Response Metric

**DOI:** 10.1289/ehp.115-1892118

**Published:** 2007-06

**Authors:** Günter Oberdörster, Eva Oberdörster, Jan Oberdörster

**Affiliations:** Department of Environmental Medicine, University of Rochester, Rochester, New York, E-mail: gunter_oberdorster@urmc.rochester.edu

Wittmaack (2007) did not agree with our suggestion (Oberdörster et al. 2005) that particle surface area is a more appropriate dose metric than particle mass or particle number when evaluating dose–response relationships of nanoparticle-induced pulmonary inflammation. According to his understanding of nanotoxicology and based on his calculations, he found particle number to work best as a dose metric. Throughout our review we pointed out that the surface area concept should be considered in the context of nanoparticle surface properties such as chemistry, charge, coating, crystallinity, porosity, and reactivity. For example, nano-titanium dioxide (TiO_2_) or nano-copper particles, very distinct from one another, will predictively create separate well-fitting surface area dose–response relationships. Yes, particle number is of importance as well, as we indicated in our review, but not as a direct dose metric.

We would like to address some of the issues Wittmaack (2007) raised in his article. First, Wittmaack suggested that when expressing a pulmonary inflammatory response, a response metric is better done using the ratio of lavaged neutrophils (PMN; polymorphonuclear leukocytes) to macrophages instead of using the fraction of PMNs. Because the purpose of our review (Oberdörster et al. 2005) was not to describe these responses in mathematical terms (whether threshold, linear, or nonlinear) but rather to illustrate that dose–response relationships on a mass basis—but not on a surface area basis—are very different, the choice of the response metric is irrelevant. To demonstrate this, we present our data again (Figure 1), expressed as absolute numbers of elicited PMNs and as PMN/macrophage ratios as a function of administered mass (Figure 1A,B), number (Figure 1C,D), or surface area (Figure 1E,F) of fine and ultrafine (nanosized) TiO_2_. The dose–response relationships based on mass and surface area are essentially the same as those shown in our review (Oberdörster et al. 2005) using the percentage of elicited neutrophils.

Second, regarding the issue of particle number being the best dose metric, the particle number dose–response relationships (Figure 1B) are several orders of magnitude apart for fine and ultrafine TiO_2_, whereas the surface area plot (Figure 1C) shows a good fit for the combined particle sizes. The reviewers of Wittmaack’s article (2007) apparently overlooked this flaw in his argument.

Finally, Wittmaack (2007) calculated that the surface area for ultrafine TiO_2_ should be 77 m^2^/g and not 50 m^2^/g, as we reported (Oberdörster et al. 2005). He derived his value on the basis of the specific density of TiO_2_ (anatase) and a spherical primary particle size of 20 nm. BET surface area for this TiO_2_ (Degussa P25) has been measured independently by a number of investigators, including our group (Jwo et al. 2005; Long et al. 2006; Wahl et al. 2005), and ranges between 48 and 55 m^2^/g. There is no reason to mathematically manipulate this number to a value that is completely at odds with actual measurements. In contrast to the well-established surface area, the average primary particle size of TiO_2_ has not been firmly established, with values of 20–30 nm. Indeed, a size of 30 nm (calculated surface area, 51.2 m^2^/g) conforms best to the measured BET surface. Thus, we added particle number dose–response data for 30 nm TiO_2_ to Figure 1C and 1D; the order of magnitude difference of the dose response between fine and ultrafine particles is obvious, regardless of whether the ultrafines are considered to be 20 or 30 nm in size.

We have concluded that of the three dose metrics discussed, particle number is the worst to describe nanoparticle-induced pulmonary inflammatory effects.

## Figures and Tables

**Figure 1:**
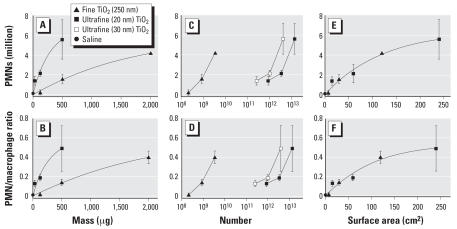
Inflammatory cell response in lung lavage 24 hr after intratracheal instillation of fine (~ 250 nm) and ultrafine (20–30 nm) TiO_2_ expressed by different dose metrics [particle mass (*A,B*), number (*C,D*), and surface area (*E,F*)] and different response metrics [number of PMNs (*A,C,E*) and PMN/macrophage ratio (*B,D,F*)].
